# Supplementary material for: Pediatric Healthcare Utilization in a Large Cohort of Refugee Children Entering Western Europe During the Migrant Crisis
Source: Int J Environ Res Public Health. 2019 Nov 11;16(22):4415. doi: 10.3390/ijerph16224415 (PMC6888204; doi:10.3390/ijerph16224415)
Supplement: Supplementary file 1 [file ijerph-16-04415-s001.pdf]

International Journal of
Environmental Research and Public Health

MDPI

Supplementary Tables and Figures

Language	%	Language	%
Arabic	41,9	Bangia	0,4
Kurdish	22,0	Tigrigna	0,3
Farsi/Dari/Persian	23,4	French	0,3
English	4,5	Bosnian	0,3
Pashto	2,5	others	0,3
Turkish	1,7	German	0,2
Albanian	0,7	Greek	0,1
Serbian	0,7	Russian	0,1
Urdu	0,5		

Supplementary Table 1. Languages spoken by refugees below the age of 18 years in the analyzed cohort.

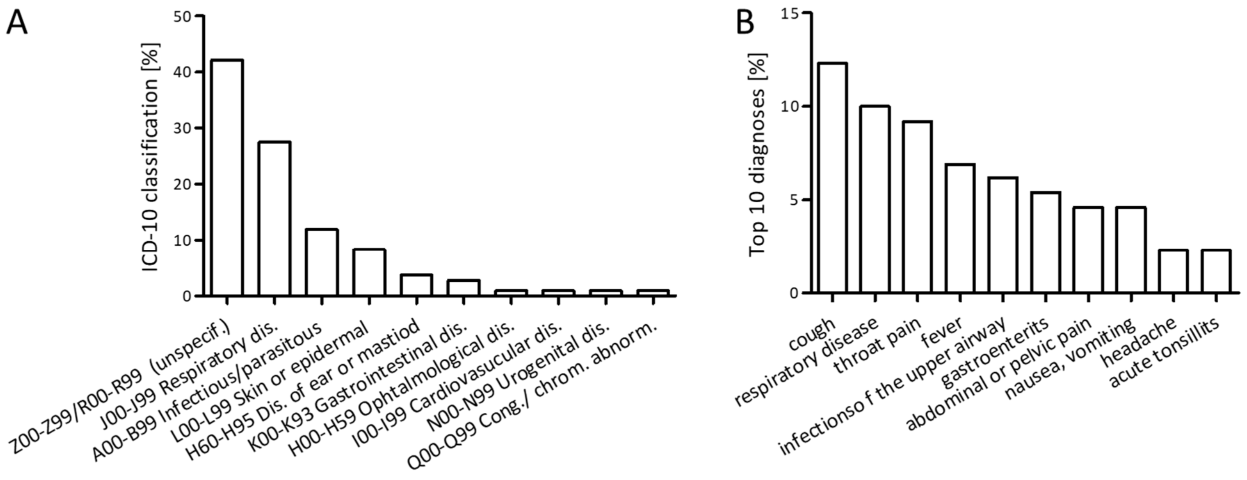

Supplementary Fig. 1. Top ten ICD-10 based diagnose groups (A) and diagnoses (B) as identified in a random sample of n=100 healthcare visits within the cohort.
